# Could smell and taste dysfunction in COVID-19 patients be a sign of the clinical course of the disease?

**DOI:** 10.1186/s43163-021-00169-8

**Published:** 2021-10-09

**Authors:** A. F. Bayrak, B. Karaca, Y. Özkul

**Affiliations:** 1 Otolaryngology-Head & Neck Surgery Clinic, Katip Celebi University, Atatürk Training and Research Hospital, Izmir, Turkey; 2 Department of Infectious Diseases and Clinical Microbiology, Katip Celebi University, Atatürk Training and Research Hospital, Izmir, Turkey

**Keywords:** COVID-19, Clinical course, Recovery, Olfactory dysfunction, Gustatory dysfunction

## Abstract

**Background:**

It could be of great benefit to determine smell and taste dysfunction in COVID-19 patients and to investigate the relationship between these symptoms and clinical characteristics as the determination of points requiring attention during the clinical course of the disease.

**Method:**

Evaluations of patients diagnosed with COVID-19 were made using a questionnaire method. Those with smell and taste dysfunction completed a visual analog scale (VAS) to determine severity. The patients were evaluated at the end of 1 and 2 months.

**Results:**

Evaluation was made of a total of 105 patients with a mean age of 55.9±17.6 years. Smell and taste dysfunction was present in 56 (53.3%) patients with a mean age of 48.7±17.6 years and the 49 (46.7%) patients with no smell and taste dysfunction had a mean age of 64±13.6 years. It was determined that as age increased, the complaints of smell and taste dysfunction decreased. Full recovery was determined in 31 (55%) patients after 1 month, and in 16 (28%) patients, smell and taste dysfunction continued at the end of the second month. The symptoms of smell and taste dysfunction were determined to last longer in patients with no comorbidities, no symptoms of fever or shortness of breath, and those treated as outpatients (*p*=0.043, *p*=0.031, *p*=0.034, *p*=0.028, respectively). In the older age patient group, the VAS scores were observed to be higher and the time to recovery was shorter (*p*=0.007, *p*=0.018, respectively).

**Conclusion:**

Smell and taste dysfunction in COVID-19 patients is seen more as age decreases and recovery takes longer. Smell and taste dysfunction is seen more in patients with symptoms of cough, nasal obstruction, and headache and lasts longer in patients without symptoms of fever and shortness of breath, with no comorbidities and in those treated as outpatients.

## Background

The ongoing COVID-19 pandemic, caused by the severe acute respiratory syndrome coronavirus 2 (SARS-CoV-2), is a cause of significant morbidity and mortality. An increasing number of patients suffer from the now well-known symptoms of loss of taste and smell [1].

The pathogenesis related to changes in smell and taste in COVID-19 patients is not yet fully understood. Changes in the sense of smell are believed to originate from damage to the olfactory bulbus or olfactory nerve caused by the virus. It has been reported that the virus causes damage directly in the oral cavity and olfactory epithelium through angiotensin-converting enzyme 2 (ACE2) receptors [2, 3].

Smell and taste dysfunction in COVID-19 patients has been confirmed with a prevalence of > 50% [4, 5]. Although many patients seem to recover the senses of taste and smell within a few weeks, these functions have been reported not to recover in a certain proportion of these patients. Although this is a low rate, it represents a large number of patients as the number of infected patients continues to increase worldwide. Moreover, there are scarce data explaining the various clinical characteristics of these symptoms, comorbidities, and the clinical course. Therefore, it is extremely important to have a good understanding of these symptoms in respect of patients regaining these sensory functions in the future and to increase their quality of life [6].

The aim of this cross-sectional study was to determine the presence of taste and smell dyfunction symptoms in patients diagnosed with COVID-19, both in-patients and outpatients, the duration of the symptoms, and the factors and clinical characteristics related to these symptoms.

## Methods

The study included patients aged > 18 years diagnosed with COVID-19 at a university hospital between 15 January and 15 March 2021 and treated either as inpatients or outpatients. The diagnosis of COVID-19 was made from PCR test positivity and/or pulmonary involvement on thorax computed tomography. Patients were excluded from the study if they had a history of olfactory dysfunction, head trauma, neurological disease, chronic rhinosinusitis, nasal polyps, or were pregnant.

Informed consent for participation in the study was obtained from each patient in compliance with the Helsinki Declaration. Approval for the study was granted by the Ministry of Health (no: T21-04-04, dated: 27-12-2020) and the hospital Ethics Committee (no: 2021-0028).

A questionnaire was created with reference to the COVID-19 Anosmia Reporting Tool developed by the American Otolaryngology-Head and Neck Surgery Academy (AAO-HNS), and patients were evaluated in respect of age, gender, comorbid diseases, and symptoms [7]. All the patients with taste and smell dysfunction were instructed to complete a Visual Analog Scale (VAS) to determine the severity. The scale was marked from 0 to 10, where 0 = no loss of senses and 10 = normal sense function. The patients were questioned about the clinical course of the disease and were re-evaluated for ongoing symptoms at the end of the first and second months.

### Statistical analysis

Data obtained in the study were analyzed statistically using SPSS vn. 24.0 software. Descriptive statistics were stated as mean±standard deviation (SD), median (minimum-maximum) values for continuous variables, and as number (*n*) and percentage (%) for categorical variables. In the examinations of univariate analyses of the loss of taste and smell, the chi-square test, Fisher, Student’s *t*, and Mann Whitney *U* tests were used as appropriate. Potential factors determined as significant in the univariate analyses were used in the multivariate logistic regression analysis performed to determine predictors. The Hosmer Lemeshow test was used for goodness-of-fit of the model. Type 1 error level of < 5% was accepted as statistically significant.

## Results

Evaluation was made of a total of 105 patients, comprising 55 males and 40 females with a mean age of 55.9±17.6 years (range, 18–91 years). Smell and taste dysfunction was present in 56 (53.3%) patients with a mean age of 48.7±17.6 years and the 49 (46.7%) patients with no smell and taste dysfunction had a mean age of 64±13.6 years.

The mean age of the COVID-19 patients with the symptom of loss of taste and smell was determined to be significantly lower than that of patients without this symptom (*p*< 0.001).

In the univariate analysis, a statistically significant correlation was determined between smell and taste dysfunction and age (*p*< 0.001), patient status as hospitalized or outpatient (*p*< 0.001), the presence of cardiological disease (p=0.017), diabetes (*p*=0.014), hypertension (*p*=0.049), a history of fever (*p*=0.017), the presence of cough symptom (*p*=0.003), shortness of breath (*p*=0.040), nasal discharge (*p*< 0.001), nasal obstruction (*p*< 0.001), muscle pain (*p*< 0.001), and headache (*p*< 0.001) (Table 1).

**Table 1:** The demographic and clinical characteristics of patients according to smell and taste dysfunction

	Loss of sense of taste and smell		
	Present (*n*, %)	Absent (*n*, %)	*P*
**Age (years)**	56 (53.3%)	49 (46.7%)	*p*< 0.001
Mean ±SD	48.7±17.6	64±13.6	
**Gender**			
Male	27 (49.1%)	28 (50.9%)	0.36
Female	29 (58.0%)	21 (42.0%)	
**Patient status**			
Hospitalized	22 (34.4%)	42 (65.6%)	< 0.001
Outpatient	34 (82.9%)	7 (17.1%)	
**Symptoms**			
Fever	25 (69.4 %)	11 (30.6%)	0.017
Cough	37 (67.3%)	18 (32.7%)	0.003
Shortness of breath	27 (65.9%)	14 (34.1%)	0.04
Muscle pain	31 (83.8%)	6 (16.2%)	< 0.001
Nasal discharge	17 (89.5%)	2 (10.5%)	< 0.001
Nasal obstruction	18 (94.7%)	1 (5.3%)	< 0.001
Abdominal pain	9 (69.2%)	4 (30.8%)	0.22
Diarrhea	12 (80.0%)	3 (20.0%)	0.025
Chest pain	9 (75.0%)	3 (25.0%)	0.11
Back pain	25 (75.8%)	8 (24.2%)	0.002
Listlessness	31 (64.6%)	17 (35.4%)	0.034
Headache	24 (82.8%)	5 (17.2%)	< 0.001
**Comorbidities**			
Smoking	9 (60.0%)	6 (40.0%)	0.57
Hypertension	10 (37.0%)	17 (63.0%)	0.049
Allergy	4 (80.0%)	1 (20.0%)	0.22
Obesity	5 (62.5%)	3 (37.5%)	0.58
Asthma	4 (66.7%)	2 (33.3%)	0.50
Cardiac disease	5 (27.8%)	13 (72.2%)	0.017
Diabetes	8 (32.0%)	17 (68.0%)	0.014
COPD	4 (40.0%)	6 (60.0%)	0.37

The independent variables observed to be significantly related to the loss of taste and smell as a result of the univariate analyses were included in the logistic regression model.

The backward method was applied in the logistic regression analysis. The beta parameters and associated *p* values, and the Exp (β) (ODDS) values are shown in Table 3. As a result of the logistic regression analysis, the effects of age and the presence of cough, nasal obstruction, and headache were determined to be significant on the emergence of the symptom of loss of taste and smell in COVID-19 patients. An increase of 1 year in age prevented 5% of the emergence of the complaint of loss of taste and smell. The presence of cough increased the risk of taste and smell dysfunction by 3.55-fold, nasal obstruction by 9.03-fold, and headache by 3.65-fold (Table 2).

**Table 2:** The results of the logistic regression analysis evaluating the risk factors for the presence of taste and smell dysfunction

			-2 Log likelihood = 102.51	
Risk factor	Beta	Standard error	OR (95% CI)	*p* value
Age (1 year)	-0.43	0.16	**0.96 (0.93–0.99)**	**0.008**
Cough symptom (+)	1.27	0.49	**3.55 (1.35–9.31)**	**0.01**
Nasal obstruction symptom (+)	2.2	1.11	**9.03 (1.02–79.9)**	**0.05**
Headache symptom (+)	1.3	0.63	**3.65 (1.07–12.5)**	**0.04**

The validity of the model obtained was assesssed with the Hosmer-Lemeshow test.

H0: The prediction equation is significant.

H1: The prediction equation is not significant.

As a result of the Hosmer-Lemeshow test, the chi-square value was calculated as 6.324. The H0 hypothesis was accepted as the model was suitable with *p* = 1.000 > α = 0.05 obtained.

At the end of the first month, the symptom of loss of taste and smell had completely recovered in 31 (55%) patients.

The VAS score for loss of smell was 1.64±2.56 at the beginning of the study, and 6.19±3.12 at the end of the second month. The VAS score for loss of taste was 2.69±2.77 at the beginning of the study, and 7.87±2.98 at the end of the second month.

A statistically significant improvement was determined in the mean VAS scores from baseline to the end of the second month (*p*=0.001), but smell and taste dysfunction persisted in 16 (28%) patients.

The mean time to recovery of the 56 patients with taste and smell dysfunction was 28.8±21.0 days (range, 5–60 days). The symptoms of taste and smell dysfunction were determined to recover in a longer period in patients with no comorbidities and those who were treated as outpatients (*p*=0.043, *p*=0.028). The time to recovery was longer in patients without the symptoms of fever and shortness of breath (*p*=0.031, *p*=0.034) (Table 3).

**Table 3:** Factors affecting the time to recovery of taste and smell dysfunction (only statistically significant factors are shown)

	Recovery of loss of sense of taste and smell (no of patients) ***n***=56	Recovery of loss of sense of taste and smell (mean days) (min-max, 5–60)	***P***
**Number of recovered patients**	31 (55%)	28.8	
**Comorbidities**			0.043
Present	36	24.5	
Absent	18	33.5	
**Fever**			0.031
Present	24	21.9	
Absent	30	34.3	
**Shortness of breath**			0.034
Present	26	34.6	
Absent	28	22.5	
**Hospitalized patients**	15	18.8	0.028
**Outpatients**	39	32.6	

In the comparisons of the VAS scores for loss of taste and smell and the mean time to recovery, it was determined that the mean VAS scores were significantly higher and the time to recovery was significantly shorter in the older age group (*p*=0.007, *p*=0.018, respectively) (Table 4).

**Table 4:** The mean time to recovery according to age groups

Age groups	Patients (***n***, %)	Mean days to recovery	Mean smell VAS score	Mean taste VAS score	***P***
18–40 years	16 (29%)	35.6	8.2	7.8	0.018
41–65 years	28 (52%)	26	8.7	9.3	
66–91 years	10 (19%)	18.5	8.9	9.4	

## Discussion

Taste and smell dysfunction was reported by 53.3% (*n*: 56) of the 105 COVID-19 patients included in this study. It was determined that as age decreased, so the occurrence of taste and smell dysfunction significantly increased. There are variable results in studies in literature that have investigated the relationship between age and taste and smell dysfunction [8]. The results of the current study showed that an increase of 1 year in age decreased taste and smell dysfunction by 5%. It was also determined that the mean VAS scores were higher and the time to recovery was shorter in the older age group. Other studies have similarly found these symptoms to be related to a younger age group [9–11].

Several studies have shown that alterations in the sense of taste and/or smell occur at the same time as other symptoms. The most common symptoms have been reported to be fever, cough, headache, and fatigue, followed by gastrointestinal symptoms at a lower rate [8]. In the current study, the presence of fever, cough, shortness of breath, nasal discharge, nasal obstruction, and headache symptoms was found to be associated with the loss of taste and smell. The likelihood of the emergence of taste and smell dysfunction symptoms was found to be 9-fold higher in the presence of nasal obstruction, and 3.5-fold higher with headache and cough. Although it has been reported in literature that there could be taste and smell dysfunction in COVID-19 patients without any other respiratory tract symptom, nasal obstruction and headache are the most frequently reported ear, nose, and throat symptoms [9, 11–14].

The pathophysiological mechanisms leading to taste and smell dysfunction in COVID-19 infection are not yet clearly understood, but they can be explained by the combination of a high viral load in the nasal mucosa and damage to the olfactory epithelium as a result of secondary inflammation. It has been reported that the virus causes damage in the oral cavity and olfactory epithelium through angiotensin-converting enzyme 2 (ACE2) receptors, and this may also result in damage to the olfactory bulb or olfactory nerve [2, 3, 15].

When the correlations were examined in this study between comorbidities and taste and smell dysfunction, it was seen that taste and smell dysfunction symptoms occurred less in patients with cardiac disease, and the time to recovery was shorter in patients with comorbidities. This result could be due to the fact that comorbid diseases are seen more in the older age group. This also supported the observations that COVID-19 patients with taste and smell dysfunction have a more mild disease course and better prognosis compared to those who do not develop taste and smell dysfunction [11, 12]. The relationship between age and loss of smell and taste is unclear, but it may be due to comorbidities in the older age group. Therefore, there is a need for further more detailed studies on this subject.

According to the questionnaire and VAS responses, 31 (55%) patients in the current study reported that taste and smell dysfunction fully recovered in mean 28 days, and in 16 (28%), the symptoms still continued at the end of the second month. Although current literature supports spontaneous recovery at mean 2–3 weeks, there are also reports of “long COVID-19” patients with symptoms lasting longer than 1 month. When it is taken into consideration that the olfactory epithelium undergoes regeneration within 6–8 weeks, it is thought that this could be another potential mechanism causing permanent anosmia. It is important to have more data related to this to clarify the pathogenesis of COVID-19 anosmia [9, 11, 16–18].

To minimize the risk of contamination and spread of COVID-19, it has been reported that taste and smell functions can be evaluated with questionnaires [19, 20]. In the current study, the questionnaire-based VAS method was used rather than objective tests, and this constitutes the main limitation of this study. Another limitation was the low number of cases. A further limitation was that intensive care unit patients could not be included as the study was questionnaire-based.

## Conclusions

Taste and smell dysfunction are symptoms which require careful evaluation in the clinical course of patients with COVID-19 infection. The fact that taste and smell dysfunction is seen in younger patients with milder clinical findings and that it can last for longer and sometimes be permanent in the younger population suggests that there is still much that is unknown related to the pathogenesis of the virus. Therefore, there is a need for further more comprehensive studies on this subject.

## Data Availability

The datasets used and/or analyzed during the current study are available from the corresponding author on reasonable request.

## Abbreviations

VAS: Visual analog scale
ACE2: Angiotensin-converting enzyme 2
AAO-HNS: American Otolaryngology-Head and Neck Surgery Academy
